# Pass-Fail Testing: Statistical Requirements and Interpretations

**DOI:** 10.6028/jres.114.013

**Published:** 2009-06-01

**Authors:** David Gilliam, Stefan Leigh, Andrew Rukhin, William Strawderman

**Affiliations:** National Institute of Standards and Technology, Gaithersburg, MD 20899

**Keywords:** binomial distribution, confidence bounds, confidence coefficient, critical value, probability of detection, probability of false alarm

## Abstract

Performance standards for detector systems often include requirements for probability of detection and probability of false alarm at a specified level of statistical confidence. This paper reviews the accepted definitions of confidence level and of critical value. It describes the testing requirements for establishing either of these probabilities at a desired confidence level. These requirements are computable in terms of functions that are readily available in statistical software packages and general spreadsheet applications. The statistical interpretations of the critical values are discussed. A table is included for illustration, and a plot is presented showing the minimum required numbers of pass-fail tests. The results given here are applicable to one-sided testing of any system with performance characteristics conforming to a binomial distribution.

## 1. Introduction

In evaluating the efficacy of equipment that is meant for detection of hidden contraband or dangerous substances, the instrument is often subjected to testing that measures its performance against requirements set forth in protocols set by national or international standards organizations. Performance requirements in these standards include those for probability of detection (PD) and probability of false alarm (PFA) at a specified level of statistical confidence.

The detection systems considered in this paper are all assumed to behave according to a binomial distribution. Only two outcomes are considered for independent trials with contraband present: the detection system either correctly reports detection or does not. Furthermore, the probability of detection must remain constant during the period of the testing. Otherwise, it may be meaning less to perform binomial model based tests to determine estimates of this quantity. Similarly, for tests with contraband absent, the detection system either correctly reports no detection, or it falsely reports the presence of contraband: and the probability of a false alarm is presumed to remain fixed throughout the period of testing.

For a detection system, PD or PFA can only be determined accurately by a sufficient number of trials. However, there is a number called the confidence level (CL) that gives some sense of adequacy of the results from a series of trials of a given size.

CL is defined in terms of the binomial probability mass function, also called the binomial discrete density function, *b*(*m*; *n*,*p*),

$$\begin{array}{c} b(m;n,p)=Pr(\text{BIN}(n,p)=m)\\ =\frac{n!}{m!(n-m)!}p^{m}{\left(1-p\right)}^{n-m}, \end{array}$$ (1)

where *m* = 0,1, …, *n*, denotes the number of successful detections or false alarms) in *n* independent trials with *p* = PD, or *p* = PFA, 0 ≤ *p* ≤ 1 (see Johnson, Kotz, and Kemp, 1992.) The number of successes in *n* repeated independent trials conforms to this function if each trial can be scored as either success or failure and the probability for success is fixed.

In Sec. 2 we discuss the definitions of CL and related critical values in detection problems. Section 3 gives statistical interpretation of these values in terms of hypothesis testing and confidence bounds. The note is concluded with Sec. 4 containing some examples.

## 2. Definitions and Test Requirements

The quantity CL can be loosely interpreted as the likelihood that any such system conforming to a binomial distribution with *m* successes in a series of *n* independent trials will have a true PD value greater or equal to a chosen value, PD*_c_*.

More formally, the accepted definition of CL in setting testing requirements is stated in terms of the equation below. The usage of this term is consonant with that of ASTM standard C 1236–99 (2005).

For a number *m* of successes found in a series of *n* pass-fail trials, with a fixed value of PD, designated PD*_c_*, the confidence level *CL*(*m*, *n*, *PD_c_*) is defined by the equation

$$CL(m,n,PD_{c})=\sum_{j=0}^{m-1}b(j;n,PD_{c}).$$ (2)

In other words, if for *x* = 0, 1, …, *n*, 0 ≤ *p* ≤ 1,

$$\begin{array}{r} \text{BINCDF}(x,n,p)=Pr(\text{BIN}(n,p)\leq x)\\ \;=\sum_{k=0}^{x}{(}_{k}^{n})p^{k}{\left(1-p\right)}^{n-k} \end{array}$$ (3)

denotes the binomial cumulative distribution function, then (2) can be expressed as

$$CL(m,n,PD_{c})=\text{BINCDF}(m-1,n,PD_{c}).$$ (4)

Note that under this definition *CL* (*m*, *n*, *PD_c_*) cannot exceed 1 − *PD^n^_c_*.

To find the critical value *m_c_*, i.e., the minimum value of *m* establishing the PD*_c_* of interest with a preselected, fixed level of confidence, CL, one must invert the inequality,

$$\text{BINCDF}(m_{c}-1,n,PD_{c})\geq CL.$$ (5)

It follows that *m_c_* is well defined only if BINCDF (*n* − 1, *n*, *PD_c_*) ≥ *CL*, i.e., if

$$1-PD_{c}^{n}\geq CL.$$ (6)

Since BINCDF(*x*, *n*, *p*) is a step-function in *x* (i.e., is not strictly increasing), it does not have a proper inverse function. If we set *m_c_* − 1, 1 ≤ *m_c_* ≤ *n* to be the least integer such that BINCDF(*m_c_* − 1, *n*, *PD_c_*) exceeds CL, then

$$m_{c}=INVBINCDF(CL,n,PD_{c})+1,$$ (7)

where *INVBINCDF*(*CL*, *n*, *p*) is the inverse cumulative binomial distribution function (i.e., is the smallest non-negative integer such that the cumulative distribution function evaluated at this value equals or exceeds CL.) Versions of this function are available in many statistical software packages, including MATLAB (*binoinv*), R (*qbinom*), NAG, GAMS, IMSL, S-PLUS, and SAS and in general spreadsheet applications, such as EXCEL (function *CRITBINOM*(*n*, *p*, *CL*).) 1

The binomial cumulative distribution function can be expressed through the incomplete beta-function,

$$\begin{array}{l} \text{BINCDF}(m-1,n,p)=1-I_{p}(m,n-m+1)\\ =\frac{\int_{p}^{1}x^{m-1}{\left(1-x\right)}^{n-m}dx}{\int_{0}^{1}x^{m-1}{\left(1-x\right)}^{n-m}dx}, \end{array}$$ (8)

*m* > 0, *n* − *m* + 1 > 0, (Abramowitz and Stegun, 1972), so that for fixed *m* and *n*, BINCDF(*m* − 1, *n*, *p*) is a decreasing function of *p*, 0 ≤ *p* ≤ 1. This formula allows one to define BINCDF(*m* − 1, *n*, *p*) for any real (non-integer) values *m* and *n* such that 0 < *m* < *n* + 1.

An analogous definition of CL applies to testing for PFA in systems where no contraband or dangerous substance is present. For any chosen value of PFA, designated PFA*_c_*, the confidence level *CL* (*m*, *n*, *PFA_c_*), equals the probability that the number of false alarms occurring in a series of *n* independent binary trials exceeds *m*. Thus, this level is defined by the equation

$$\begin{array}{c} CL=CL(m,n,PFA_{c})=\sum_{k=m+1}^{n}b(k;n,PFA_{c})\\ =1-\text{BINCDF}(m,n,PFA_{c}). \end{array}$$ (9)

Similarly to the PD case,

$$CL\leq 1-{\left(1-PFA_{c}\right)}^{n}.$$ (9)

To find the maximum value *M_c_* of *M*, *M* = 0, 1, …, *n* − 1, establishing the PFA*_c_* of interest with a preselected, fixed level of confidence CL, one must invert the inequality

$$1-\text{BINCDF}(M_{c},n,PFA_{c})\geq CL.$$ (11)

To express *M_c_* through the function INVBINCDF (*c*, *n*, *p*), i.e., to establish the largest value *m* satisfying (11), the formula,

$$\begin{array}{l} \text{INVBINCDF}(c,n,p)=n=1\\ -\max\{x:\text{BINCDF}(x,n,1-p)\leq 1-c\}, \end{array}$$ (12)

can be employed. To prove (12), notice that for *x* = 0, …, *n* − 1,

BINCDF(x,n,p)=1−BINCDF(n−x−1,n,1−p), (13)

so that

$$\begin{array}{l} n-1-\text{INVBINCDF}(c,n,p)\\ =n-1-\min\{x:\text{BINCDF}(x,n,p)\geq c\}\\ =n-1-\min\{x:\text{BINCDF}(n-x-1,\;n,1-p)\leq 1-c\}\\ =\max\{x:\text{BINCDF}(x,n,1-p)\leq 1-c\}. \end{array}$$ (14)

Therefore,

$$M_{c}=n-1-\text{INVBINCDF}(c,n,1-PFA_{c}),$$ (15)

so that *M_c_* ≤ *n* − 1 and *M_c_* is not defined when

$$INVBINCDF(CL,n,1-PFA_{c})=n,$$

i.e., when (1 − *PFA_c_*)*^n^* > 1 − *CL*.

Thus (15) and (7) show that under the same value of CL, when PD = 1− PFA, a simple formula,

$$m_{c}+M_{c}=n,$$ (16)

relates *m_c_* and *M_c_*.

## 3. Hypothesis Testing and Confidence Bounds on Binomial Probability

We give here two statistical interpretations of Eq. (7) and Eq. (15). The first of these is related to a (lower) confidence limit for binomial probability *p*. Such limits are supposed to provide a data-dependent interval containing the unknown *p* with a given probability called *confidence coefficient* (see Hahn and Meeker, 1991).

Assume that for the given CL, a lower confidence bound for PD = *p* of confidence coefficient CL is desired: that is for a binomial observation *X* ~ *BIN* (*n*, *p*), one requires a function $\underline{p}=\underline{p}\;(X,n,\text{CL})$ such that

$$Pr(\underline{p}(X,n,CL)\leq p)\geq CL.$$ (17)

The well known solution of this problem for *X* ≥ 1, is

p(X,n,CL)=max{p:BINCDF(X−1,n,p)≥CL}. (18)

(e.g, Casella and Berger, 2002.) When *X* = 0, $\underline{p}(0,n,CL)=0$.

Thus with *mc* defined by (7), the inequalities $\underline{p}<p$ (strict inequality) and *X* ≤ *m_c_* (non-strict inequality) are equivalent. Therefore, the critical value *m_c_* has the interpretation of the largest value of the binomial *BIN*(*n*, *p*) variable such that the lower confidence bound for *p* does not exceed *PD_c_*.

A related interpretation is provided by the statistical hypothesis testing problem, *H*_0_ : *p* ≥ *PD_c_* under the alternative: *H*_1_ : *p* < *PD_c_*. The most powerful test of level 1 − CL rejects *H*_0_ when the observed value *X* exceeds the critical value *m*, *X* > *m* (which means the same as $\underline{p}(X,n,CL)\geq PD_{c})$.

The critical value for PFA has a similar statistical interpretation, namely, *M_c_* is the largest value of the binomial variable for which the upper confidence bound for the binomial probability does not exceed *PFA_c_*. Indeed, an upper confidence bound of confidence coefficient CL has the form,

$$\bar{p}(X,n,CL)=1-\underline{p}(n-X,n,CL).$$ (19)

Identity (13) shows that

$$\bar{p}(X,n,CL)=\min\{p:\text{BINCDF}(X,n,p)\leq 1-CL\}.$$ (20)

$$\begin{array}{l} \text{Thus},\;\bar{p}(M_{c},n,CL)\leq PFA_{c},\\ \text{but}\;\bar{p}(M_{c}+1,n,CL)>PFA_{c}. \end{array}$$

In terms of the hypothesis testing with *H*_0_ : *p* ≤ *PFA_c_* and the alternative: *H*_1_ : *p* > *PFA_c_*, the most powerful test of level 1-CL rejects *H*_0_ when the observed value *X* exceeds the critical value *M_c_*, *X* > *M_c_*.

## 4. Examples

Consider an example in which one finds twenty-nine correct results in a single set of thirty trials. If the system under test conforms to a binomial distribution, then based on the result of twenty-nine out of thirty correct responses in that one set of tests, one can make multiple correct inferences, such as: the PD > 0.95 with 44 %, confidence, the PD > 0.90 with 81 %, confidence, or the PD > 0.85 with 95 % confidence.

One can easily construct a table which simultaneously includes requirements for both PD and PFA.

Table 1 gives the critical value *M_c_* and *n* − *m_c_* for 68 % confidence to show the general characteristics of these quantities. These are the maximum permissible numbers of incorrect results that may be tolerated in establishing the specified PD or PFA values at this level of confidence. If the tabulated value is indicated as “*”, then the number of trials in that set is insufficient to establish the corresponding PD or PFA at this confidence level. One may generate tables of this kind for any CL, PD, and PFA using Eq. (7) and Eq. (15) by using the previously mentioned functions like *binoinv* or *CRITBINOM* from statistical software packages or spreadsheet applications. The actual value of *M_c_* and *n* − *m_c_* given by these functions in the cases marked by “*” is − 1.

The symmetry of testing requirements when PFA = 1 − PD permits tabulating the results for PFA and PD in a single table, but it does not imply that PFA should or must always be chosen equal to 1 − PD. The PD and PFA values may be assigned independently in any testing protocol. In fact, to avoid disruption of the stream of commerce by large numbers of false alarms, it is often necessary to require inspection equipment to have PFA smaller than 1 − PD.

By solving (6) or (10), we obtain a formula for the minimum number of required trials *n_k_* needed to establish a given value of PD or PFA for the same *CL*,

$$n_{k}=\left\lceil a\right\rceil ,$$ (21)

with

$$a=\frac{\log(1-CL)}{\log PD}=\frac{\log(1-CL)}{\log(1-PFA)}.$$ (22)

**Fig. 1:**
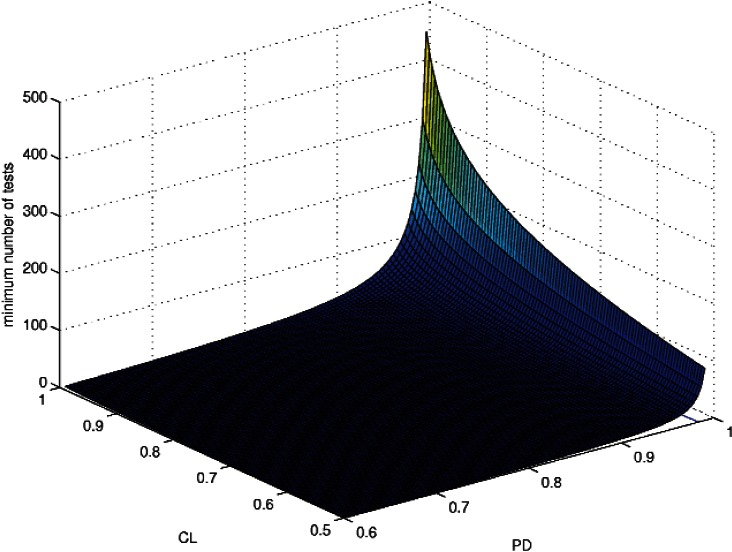
The minimum required number of tests to establish a given value of PD (or 1-PFA) for a given CL.

Here ⌈a⌉ denotes the smallest integer exceeding *a*. This formula is useful in designing test protocols that give the most satisfactory requirement with the least amount of testing. Figure 1 shows *a* plotted as a function of PD and CL. This function increases much more rapidly for *PD* approaching 1 than for *CL* → 1. Similarly *n_k_* in (21) would increase much more rapidly for *PFA* → 0 than for *CL* → 1.

When only the minimum number of trials *n_k_* is performed, the system must give 100 % correct results to establish the specified PD or PFA at, the desired confidence *CL*. In statistical terms, *n_k_* is the smallest number of trials with 100 % correct detections such that the *CL*-lower confidence bound for detection probability exceeds the given value PD. The same is true when there are no false alarms with the *CL*-upper confidence bound on the false alarm probability being less than PFA. A table such as Table 1 will show how many errors may be permitted if a larger number of trials are carried out, while still establishing the specified PD or PFA at the desired CL.

## 5. Discussion and Conclusions

The formula for *n_k_* shows that requiring either PD or CL to be too near unity can result in impossibly large numbers of pass-fail tests. If such rigorous criteria are in fact required then one should search for some method of verification different from pass-fail testing.

The results presented here make it possible to design pass-fail testing protocols based on functions readily available in statistical software packages and general spreadsheet applications.

## Figures and Tables

**Table 1:** Maximum permissible numbers of incorrect results for verifying a lower bound on PD or an upper bound on PFA with 68 % confidence

PD→	0.95	0.90	0.85	0.80	0.75	0.70	0.60	0.50

PFA→	0.05	0.10	0.15	0.20	0.25	0.30	0.40	0.50
n = 2	*	*	*	*	*	*	*	0
n = 3	*	*	*	*	*	*	0	0
n = 4	*	*	*	*	0	0	0	1
n = 5	*	*	*	*	0	0	0	1
n = 6	*	*	*	0	0	0	1	1
n = 7	*	*	*	0	0	0	1	2
n = 8	*	*	0	0	0	1	2	2
n = 9	*	*	0	0	1	1	2	3
n = 10	*	*	0	0	1	1	2	3
n = 11	*	0	0	0	1	2	3	4
n = 12	*	0	0	1	1	2	3	4
n = 13	*	0	0	1	1	2	3	5
n = 14	*	0	0	1	2	2	4	5
n = 15	*	0	1	1	2	3	4	6
n = 16	*	0	1	1	2	3	4	6
n = 17	*	0	1	2	2	3	5	7
n = 18	*	0	1	2	3	3	5	7
n = 19	*	0	1	2	3	4	6	7
n = 20	*	0	1	2	3	4	6	8
n = 21	*	0	1	2	3	4	6	8
n = 22	*	0	1	2	3	5	7	9
n = 23	0	1	2	3	4	5	7	9
n = 24	0	1	2	3	4	5	7	10
n = 25	0	1	2	3	4	5	8	10
n = 30	0	1	2	4	5	7	10	13
n = 40	0	2	4	6	8	10	14	18
n = 50	1	3	5	8	10	12	17	22
n = 60	1	4	7	9	12	15	21	27
n = 70	2	5	8	11	15	18	25	32
n = 80	2	6	9	13	17	21	29	37
n = 90	2	7	11	15	20	24	33	42
n = 100	3	7	12	17	22	27	37	47

## Footnotes

1 Any mention of specific commercially available statistical software packages or general spreadsheet applications does not imply endorsement of preference for these products by the NIST.
